# Immunoinflammation and post-translational modifications in the aging process

**DOI:** 10.1186/s12967-025-06892-7

**Published:** 2025-08-14

**Authors:** Jiaqi Xiao, Xuan Qin, WenTao Chen, Xinyu Que, Yaoyao Ma, Wentao Huang, Haoxiang Ou, Yongfen Bao, Lihua Qu, Shigang Shan

**Affiliations:** 1 Hubei Key Laboratory of Diabetes and Angiopathy, School of Pharmacy, Hubei University of Science and Technology, Xianning, 437000 China; 2 School of Basic Medical Sciences, Xianning Medical College, Hubei University of Science and Technology, Xianning, 437000 China; 3 Department of Pharmacy, Ezhou Central Hospital, Ezhou, 436000 China; 4 School of Basic Medical Sciences, Hunan Normal University, Changsha, 410013 China

**Keywords:** Aging, Immunosenescence, Inflammaging, Histone modification, Post-translational modification

## Abstract

Aging is an intrinsic biological decline marked by multidimensional alterations spanning molecular, cellular, tissue, and organ levels. One hallmark of aging is the progressive deterioration of immune function, a condition referred to as immunosenescence. This process often involves a persistent, mild, and non-infectious inflammatory state across the body, commonly described as inflammaging. The regulation of age-related immune and inflammatory processes is critically influenced by epigenetic mechanisms, such as alterations in DNA methylation patterns, histone modifications, chromatin structure reorganization, and the regulatory actions of non-coding RNAs. Recent research has increasingly focused on the regulatory roles of post-translational modifications (PTMs), including histone methylation, acetylation, ubiquitination, and O-GlcNAcylation, have been widely recognized as fundamental modulators of immunoinflammatory processes in aging. In this review, we provide a comprehensive overview of histone modification-mediated mechanisms involved in the regulation of immunosenescence. We further highlight their functional roles from the perspective of immune inflammation and explore potential therapeutic strategies targeting histone modifications to mitigate immunosenescence.

## Introduction

Aging is an intrinsic biological decline marked by multidimensional alterations spanning molecular, cellular, tissue, and organ levels. Recent studies have identified aging as a multifaceted biological process influenced by key hallmarks such as cellular senescence, genomic instability, telomere shortening, epigenetic changes, proteostasis collapse, dysregulated nutrient sensing, mitochondrial impairment, stem cell depletion, and disrupted intercellular signaling [1, 2]. Notably, the aging process emerges as the predominant determinant for the development of chronic disorders, declines in both physical and mental faculties, and increased susceptibility to mortality [3]. The gradual deterioration of homeostatic capacity with advancing age renders organisms increasingly vulnerable to age-associated pathologies (e.g., cardiovascular diseases, cancer, and neurodegenerative disorders) and substantially elevates mortality risk [4].

The decline in immunological competence represents a key biological consequence of aging, a phenomenon scientifically termed immunosenescence. A defining feature of this process involves the structural degeneration and reorganization of immune organs, coupled with declining efficacy in both nonspecific and antigen-specific immune mechanisms. This decline results in suboptimal vaccine efficacy and heightened vulnerability to infections and age-associated pathologies [5]. The hallmark features of immunosenescence include diminished immune responsiveness coupled with an exacerbated proinflammatory status [6]. This paradoxical phenomenon, designated as inflammaging (inflammatory aging), is considered a consequential manifestation of immunosenescence [7] and acts as a pivotal driver underlying the progression of age-associated pathologies.

**Fig. 1:**
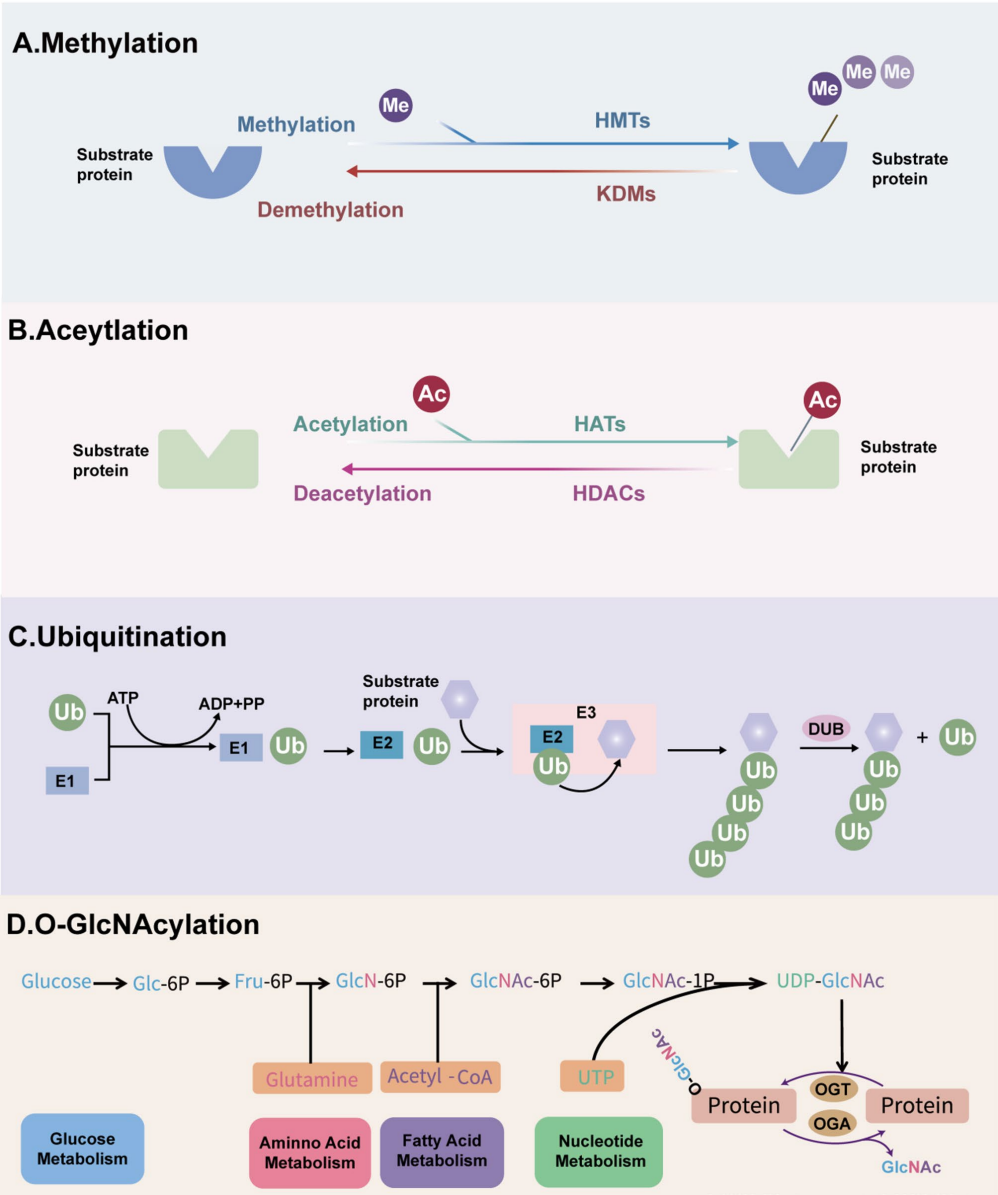
Common histone PTMs processes. **A** Histone methylation is dynamically regulated by HMTs and KDMs. **B** Histone acetylation is modulated by HATs and HDACs. **C** In ubiquitination, ubiquitin-activating enzyme (E1) activates Ub in an ATP-dependent manner, which is then transferred to a ubiquitin-conjugating enzyme (E2), forming a ubiquitin–E2 complex. The ubiquitin ligase (E3) facilitates the transfer of Ub from the E2 enzyme to the substrate protein. This modification can be reversed by DUBs. **D** O-GlcNAcylation is primarily regulated via the hexosamine biosynthetic pathway (HBP), which integrates four major metabolic pathways: glucose metabolism (glucose), amino acid metabolism (glutamine), fatty acid metabolism (acetyl-CoA), and nucleotide metabolism (UTP). OGT and OGA control the addition and removal of O-GlcNAc, respectively

In eukaryotes, epigenetic modifications play pivotal roles in transcriptional regulation [8]. As a fundamental mechanism of gene expression control, epigenetics is characterized by reversible chromatin-based regulatory processes that modulate gene activity while maintaining the DNA sequence [9]. The epigenome serves as a critical interface between genotype and phenotype, dynamically responding to environmental stimuli and orchestrating phenotypic plasticity during aging [4]. Major epigenetic regulatory mechanisms include histone modifications, DNA methylation, chromatin remodeling, and non-coding RNA-mediated regulation.Importantly, aging does not affect all components of the immune system equally, and epigenetic regulation serves as essential modulators of shaping immune cell identity and function [10]. Emerging research on post-translational modifications (PTMs), notably histone methylation, acetylation, ubiquitylation, and O-GlcNAcylation (Fig. 1), has elucidated their specialized roles in modulating immune-inflammatory responses during senescence.

During immunosenescence, PTMs reshape the immune microenvironment through epigenetic regulation and signal transduction, profoundly influencing the senescence-associated secretory phenotype (SASP), autophagy, and the perpetuation of chronic inflammation. Histone methylation, predominantly occurring at H3K4, H3K9, H3K27, and H3K36 residues, is catalyzed by methyltransferases such as KMT2A, SUV39H1, and EZH2. These enzymes modulate gene activation, autophagic processes, and transcriptional silencing, thereby regulating cellular senescence and inflammatory responses [11]. Acetylation modifications, including H3K9ac, H3K27ac, and H4K16ac, are dynamically regulated by histone acetyltransferases (HATs) and histone deacetylases (HDACs). These modifications facilitate gene expression and telomeric silencing, ultimately influencing transcriptional activity and immune function [12]. Ubiquitination, mediated by E3 ubiquitin ligases, targets histones and associated proteins, modulating proteasomal degradation, mitophagy, and inflammatory signaling pathways. This regulatory mechanism is critical for maintaining proteostasis during aging [13–15]. The non-canonical O-GlcNAcylation, catalyzed by O-GlcNAc transferase (OGT), modifies serine and threonine residues on histones. This modification enhances the activity of antioxidant transcription factors and suppresses aberrant tau phosphorylation in neurodegenerative disorders, thereby supporting cognitive function and immune defense mechanisms [16, 17]. Collectively, these PTMs orchestrate cellular responses to senescence-inducing stimuli by fine-tuning chromatin architecture and gene expression, positioning them as pivotal therapeutic targets in immunosenescence and related pathologies.

In this review, we begin by outlining the concept and defining features of immunosenescence, along with the aging-associated alterations observed across various immune cell populations. Subsequently, we elucidate the mechanistic links between immunosenescence and the pathogenesis of age-dependent diseases. Furthermore, we synthesize recent discoveries elucidating how specific histone modifications in immune aging. Finally, we critically evaluate emerging therapeutic strategies targeting histone-modifying pathways to counteract immunosenescence.

## Immunosenescence: concept and characteristics

**Fig. 2:**
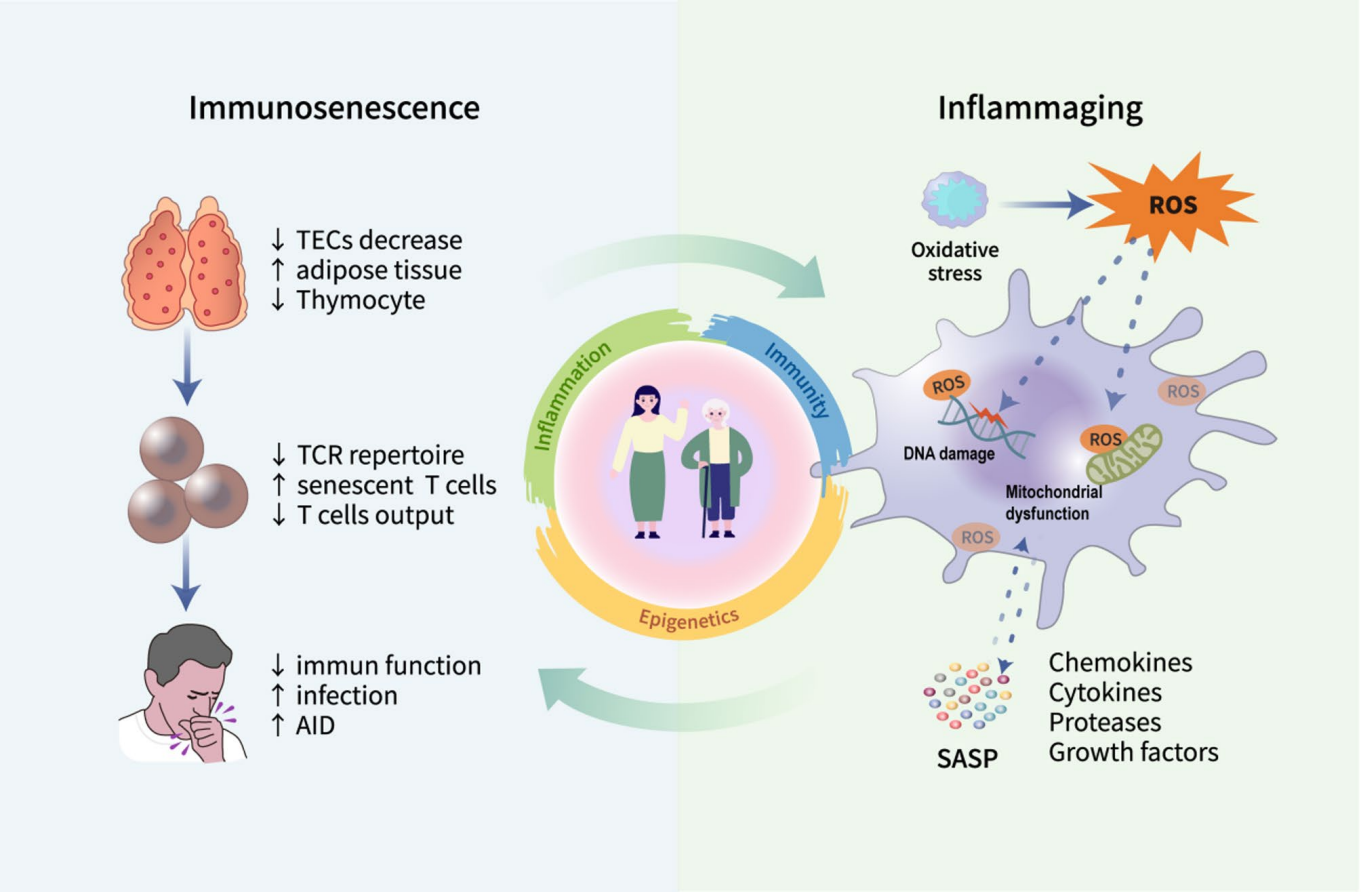
Characteristics of immunosenescence. Immunosenescence is driven by multiple factors, including thymic involution, diminished T cell output, and the accumulation of SASP induced by inflammaging. Senescent immune cells exhibit impaired ability to clear both senescent cells and inflammatory mediators, thereby reinforcing a detrimental cycle between inflammaging and immunosenescence

Immunosenescence describes the progressive decline of immunological competence associated with aging [18]. This process involves multifaceted changes in immune cell populations—such as T cells, B cells, and macrophages—leading to compromised immunity in older adults. Consequently, they face higher risks of infections, autoimmune disorders, and malignancies [19, 20]. Immunosenescence is characteristically manifested by thymic involution, T-cell depletion, and inflammaging(Fig. 2). The thymus is a crucial immunological structure, facilitating the generation and maturation of immune cells, particularly for T lymphocytes [21]. Thymic atrophy represents the most prominent hallmark of immunosenescence [22]. Histologically comprising epithelial components and perivascular non-epithelial elements, the thymus undergoes profound age-associated architectural changes. The pathological features include: (1) progressive loss of thymic epithelial cells (TECs), (2) ectopic adipose tissue accumulation, and (3) developmental arrest of thymocytes [23]. These alterations collectively impair naïve T-cell output and reduce peripheral T-cell receptor (TCR) diversity, ultimately compromising immune competence and predisposing to chronic low-grade inflammation [24]. Notably, patients with congenital heart disease (CHD) who underwent thymectomy exhibited accelerated immunosenescence, characterized by persistent T-cell lymphopenia and diminished naïve T-cell pools [25]. These findings highlight thymic rejuvenation as an effective intervention to mitigate immunological deterioration associated with aging.

Moreover, immunosenescence is often accompanied by a persistent, mild, and non-infectious inflammatory state across the body, a process termed “inflammaging,” which is now identified as a defining feature of aging [2]. Inflammaging arises from the intricate interaction between increased pro-inflammatory mediators and impaired immune cell function, leading to widespread dysregulation of both the innate and adaptive immune systems, and ultimately resulting in a diminished immune response to antigenic stimuli [26]. One proposed mechanism underlying inflammaging is the preferential preservation of innate immune responses, which tend to remain relatively intact with age, in contrast to the progressive decline in adaptive immunity. This imbalance may foster excessive pro-inflammatory activity, thereby accelerating the inflammaging process [27]. Additionally, oxidative stress, which increases during aging, exacerbates this pathological state, amplifying chronic inflammatory processes [28]. Evidence indicates that this persistent inflammatory state stems from chronic oxidative stress induced by sustained immune activation [29]. The bidirectional interplay between oxidative stress and inflammation forms the foundation of the so-called “oxi-inflamm-aging theory” of aging [30, 31]. Persistent oxidative stress induces excessive reactive oxygen species (ROS) production by immune cells, bringing about cellular damage, enhanced recruitment of inflammatory cells, and a self-perpetuating loop of inflammatory amplification and oxidative injury [32]. High ROS exposure causes DNA damage, mitochondrial dysfunction, inducing senescence in cells accompanied by the emergence of the SASP [33]. SASP is composed of various chemokines, cytokines, proteases, and growth factors that mediate detrimental paracrine effects on proximate cells, disrupt extracellular matrix composition, and impair tissue ultrastructure [34]. The sustained secretion of SASP factors is thus a major driver of inflammaging.Although immune cells serve as the primary surveillance and clearance mechanism for senescent cells, SASP factors may impair immune surveillance, leading to the accumulation of senescent cells and chronic inflammatory signaling molecules, thereby creating a vicious cycle between inflammation and aging [35]. Moreover, cellular senescence, mitochondrial dysfunction, DNA damage, and alterations in the gut microbiota have all been closely implicated in the progression of inflammaging and may act synergistically to exacerbate it [28, 36]. In conclusion, regardless of the specific nature or contribution of each component involved, inflammaging ultimately leads to a persistent subclinical inflammatory status. Whether early intervention in inflammaging can effectively prevent or delay the onset of cellular senescence, immunosenescence, and related age-associated diseases remains an important question that warrants further validation in clinical studies.

## Alterations in immune cell populations during Immunosenescence

**Fig. 3:**
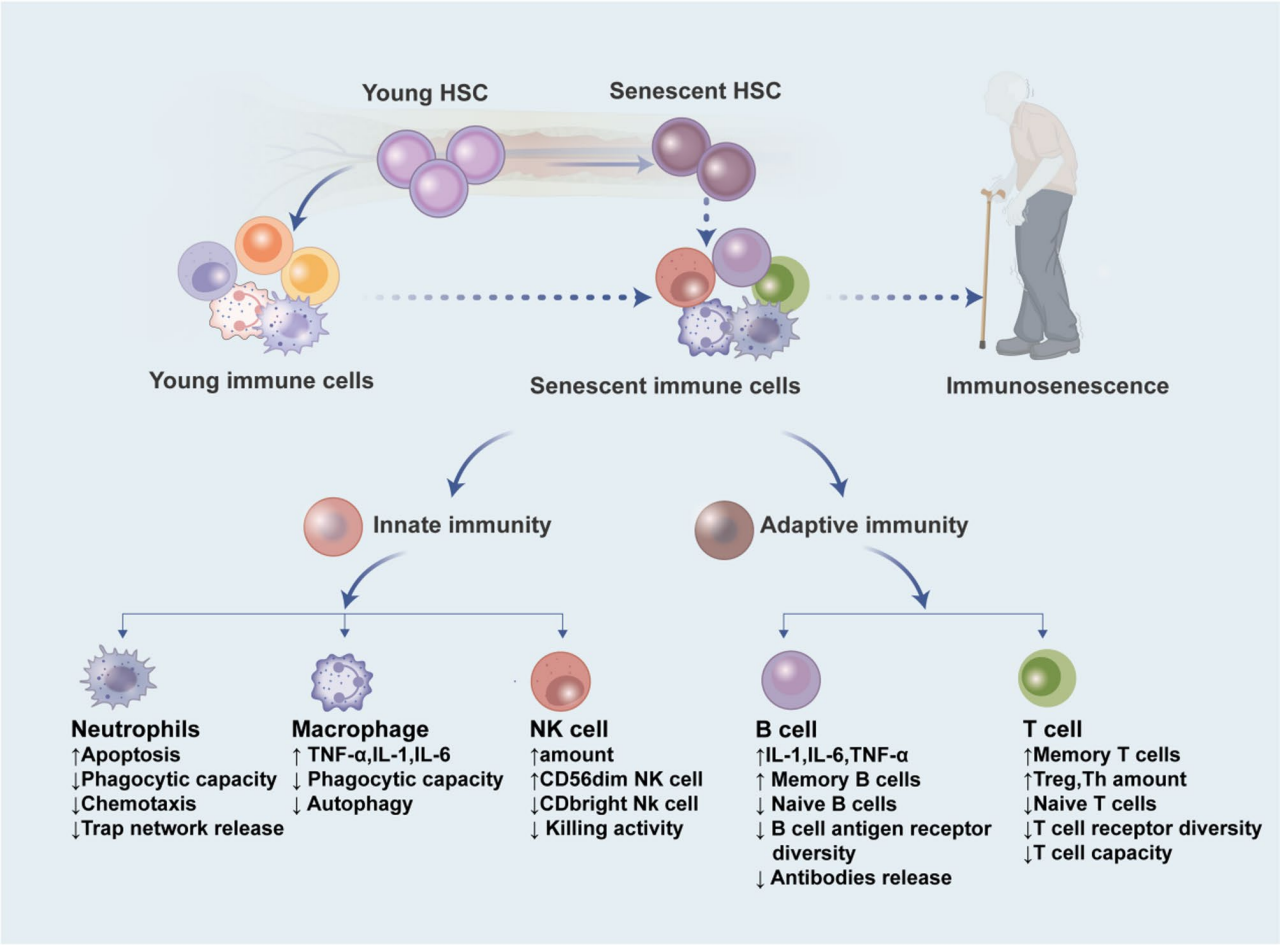
Alterations of immune cells during immunosenescence. Immunosenescence leads to both quantitative and functional changes in various immune cell types, including neutrophils, macrophages, NK cells, B cells, and T cells

Immunosenescence manifests through progressive deterioration of immune components, encompassing both innate and adaptive immunity systems [37, 38]. The innate arm primarily involves neutrophils, natural killer (NK) cells, monocytes, macrophages and dendritic cells, whereas the adaptive system mainly comprises T and B lymphocytes [39]. This age-related immunological decline constitutes a multifactorial cascade process, with heterogeneous susceptibility observed across distinct immune cell populations [40]. Consequently, immunosenescence serves as a critical role in cellular subset homeostasis(Fig. 3).

### Neutrophils and Immunosenescence

Representing approximately 70% of circulating leukocytes [41], neutrophils serve as the primary cellular mediators of innate immune defense in humans. Their key immune functions include phagocytosis of pathogens, degranulation (release of antimicrobial factors), reactive ROS production, and neutrophil extracellular trap (NET) formation [42]. However, these functions decline significantly with aging [43]. In murine sepsis models, neutrophils from aged individuals exhibited reduced recruitment efficiency and impaired phagocytic capacity relative to young controls [44, 45]. A similar age-dependent decline in chemotaxis and pathogen clearance has been revealed in older individuals with community-acquired pneumonia (CAP) [46]. This functional deterioration of neutrophils might have compromised early immune defense, exacerbating infection progression. Furthermore, polymorphonuclear leukocytes (PMNs) from older individuals exhibited reduced phagocytic activity, accompanied by an increase in the proportion of immunosuppressive PMN subsets (CD16bright/CD62Ldim), leading to decreased ROS levels and phagocytic index. These alterations significantly increased susceptibility to bacterial and fungal infections [47]. NETs, a unique extracellular meshwork composed of DNA, histones, and granular proteins, play a crucial role in pathogen trapping and killing [48]. However, in HIV-infected individuals, aging impaired calcium signaling and TLR8 activation in blood and genital tract neutrophils, suppressing NET release. This dysfunction weakened anti-HIV immune responses, potentially increasing HIV infection risk in women [49].

Age-related defects in neutrophil chemotaxis were closely associated with increased PI3K activity. Studies demonstrated that pharmacological inhibition of PI3K effectively restored chemotactic function in neutrophils from aged individuals [50]. Immunosenescence also profoundly alters neutrophil migratory behavior [51]. Barkaway et al. [52] revealed that aging elevated mast cell-derived CXCL1, promoting reverse transendothelial migration (rTEM) of neutrophils in inflamed tissues. This aberrant process led to neutrophils re-entering circulation and subsequent migration to distal organs, exacerbating vascular leakage and remote organ damage.Similarly, Rolas et al. [53] demonstrated that progerin-expressing endothelial cells (ECs) enhance CXCL1 production, driving pathological neutrophil recruitment to inflamed tissues and amplifying tissue injury and inflammatory responses.

### NK cells and immunosenescence

Serving as primary cytolytic executors in innate immune responses, NK cells are crucial for recognizing and eliminating various compromised cells, particularly virus-infected cells and cancerous cells [54, 55]. Intriguingly, although overall immune cell levels decline with aging, NK cell populations exhibit a paradoxical expansion [56, 57]. However, in certain malignancies, NK cell abundance is markedly reduced. For instance, studies in non-small cell lung cancer (NSCLC) laboratory mouse paradigms revealed significantly diminished NK cell infiltration in geriatric relative to juvenile murine subjects [58]. Similarly, hepatocellular carcinoma (HCC) patients demonstrated a notable reduction in NK cell infiltration within the tumor microenvironment (TME), with this decline strongly correlating with advancing age and age-associated phenotypic changes [59]. In endometrial cancer, dysregulation of key chemokines (CXCL12, IP-10, and CCL27) and cytokines (IL-1β and IL-6) within the TME impaired NK cell functionality and recruitment, suggesting that the TME actively remodeled NK cell characteristics to support cancer progression [60]. Furthermore, aging influenced NK cell subtypes, with aged individuals exhibiting decreased proportions of CD56bright NK cells and increased CD56dim populations [56, 61]. Phenotypic shifts also occured during aging; Deng et al. identified an elevated percentage of PD-1⁺ NK cells in older individuals, which co-expressed CD57 and CD69, revealing that PD-1⁺ NK cells represented a senescence-associated immunophenotype [62]. Mechanistically, Chen et al. reported that Vps34 (the sole class III PI3K family member) deficiency impaired autophagy in terminally differentiated NK cells, leading to ROS accumulation and a premature senescence phenotype—a process reversible upon antioxidant intervention [63]. Functionally, age-related NK cell dysfunction manifested as reduced cytotoxicity, diminished secretion of key cytokines (e.g., IL-2 and IL-12), and consequently, compromised immune surveillance, increasing susceptibility to infections, oncogenesis, and mortality. Notably, visceral adipose tissue (VAT)-derived IL-15 and its receptor IL-15Rα may partially mitigated these detrimental effects [57, 64].

Fortunately, emerging therapeutic strategies have shown promise in counteracting aging-induced NK cell dysfunction. For instance, melatonin enhanced NK cell maturation and activation by stimulating the JAK3/STAT5 pathway, upregulated T-bet expression, and consequently augmented NK cell proliferation, degranulation, and IFN-γ secretion—offering a potential immunomodulatory approach for the elderly [65]. Another intervention, arabinoxylan rice bran (Biobran/MGN-3), boosted NK cell cytotoxicity by upregulating CD107a expression, potentially improving elderly individuals’ immune defense against oncological and viral challenges [66]. Similarly, Lactobacillus plantarum HY7712, a probiotic strain derived from kimchi, mitigated radiation- or senescence-associated NK cell impairment via the TLR2/NF-κB pathway [67]. More definitively, NK cell adoptive transfer has demonstrated efficacy in rejuvenating immunosenescent systems, clearing senescent cells (SNCs) from tissues, and reducing SASP factors—highlighting its therapeutic potential for age-related pathologies [68–70].

### Macrophages and immunosenescence

Macrophages are found in nearly every organ system and are essential for immune homeostasis, thanks to their exceptional phagocytic capacity, complement component recognition, and antigen-presenting functions [71]. However, aging induces significant alterations in macrophage phenotype and functionality, characterized by diminished phagocytosis and chemotaxis, impaired autophagy, and heightened production of pro-inflammatory cytokines alongside excessive reactive ROS release [72]. For instance, upon LPS stimulation, macrophages derived from elderly individuals exhibited markedly increased secretion of IL-1, TNF-α, and IL-6 relative to those from younger subjects [73]. Vida et al. [74] further demonstrated that macrophages from older mice accumulated greater quantities of ROS and lipofuscin, concomitant with functional impairment. These results highlighted macrophages’ pivotal involvement in age-associated chronic oxidative stress, positioning them as key contributors to immunosenescence and the “oxidative-inflammatory aging” axis [75]. Additionally, the decline in both abundance and rhythmicity of Krüppel-like factor 4 (Klf4) in aged macrophages disrupted circadian regulation, subsequently attenuating immune responsiveness [76]. Such functional decline exacerbated chronic inflammation and tissue degeneration, predisposing to multiple age-related pathologies. Pappert et al. [77] identified that senescent macrophages released cytotoxic mediators, accelerating osteoporosis. Similarly, age-altered dermal macrophages drove adaptive immune dysfunction, fostering pro-inflammatory microenvironments conducive to conditions like skin cancer [78]. Immunosenescence may also impair hyalocytes (vitreous-resident macrophages), thereby promoting vitreoretinal disorders [79]. Notably, Liu et al. [80] observed an elevated proportion of senescent macrophages in murine muscular dystrophy models. Treatment with the senolytic agent fisetin reduced senescent macrophage burden, restored myocyte populations, and improved muscle phenotype, suggesting that senescent macrophages inhibited muscle stem cell function and contributed to dystrophic progression—highlighting fisetin as a potential therapeutic strategy.Mechanistically, deficient type 2 cytokine signaling accelerated macrophage aging, whereas the IL-4–STAT6 axis conferred protection against senescence [81]. Furthermore, T-2 toxin triggered immunosenescence in RAW264.7 cells via cGAS-STING pathway activation, implicating this pathway as a potential therapeutic target [82]. Autophagy, an intracellular degradation process linked to longevity, critically regulated macrophage homeostasis. Atg7-deficient macrophages exhibited metabolic shifts toward glycolysis, accompanied by hallmark senescent phenotypes: proliferative arrest, loss of phagocytic function, and hyperinflammatory cytokine responses [83]. Thus, autophagy serves as a guardian against immunosenescence by sustaining metabolic flexibility and suppressing inflammatory cascades.

### T-cell and immunosenescence

The aging process triggers profound structural and functional remodeling across immune cell subsets, encompassing comprehensive adaptations in cellular phenotypes and physiological activities. Of particular interest are T lymphocytes, the central effectors of cellular immunity, whose dynamic evolution during aging has attracted significant research attention [84]. Immunosenescence is marked by the quiescent persistence of naïve and memory T cells, which retain the potential for rapid activation, proliferation, and differentiation upon antigenic challenge [85, 86]. Notably, age-related decline in thymic output capacity emerges as a hallmark of immunosenescence, concomitant with elevated SASP, enhanced glycolytic flux, and reactive oxygen species accumulation [18]. Concurrently, elderly individuals exhibit reduced pools of proliferative naïve B/T cells, constricted receptor repertoire diversity, and amplified pro-inflammatory senescent secretory profiles. Furthermore, aging impairs germinal center reactions and compromises the architecture/function of secondary lymphoid organs, resulting in dysregulated T-B cell dynamics and increased autoreactivity [87].

Senescent T cells may propagate inflammatory cascades through direct cell-cell contact, paracrine cytokine signaling, or tissue-targeting effects, ultimately driving tissue damage and contributing to aging pathogenesis [88]. Intriguingly, B cells serve as a pivotal regulator of T cell fate determination, with studies demonstrating B cells’ involvement in age-associated naïve T cell loss through modulation of TCR clonal expansion and pathogenic T subset differentiation [89]. Aging also fosters the accumulation of specialized T cell subsets, including: (i) highly cytotoxic NK-like populations, (ii) cytokine-dysregulated exhausted subsets, and (iii) pro-inflammatory regulatory T cells. These populations share impaired lymphoid homing capacity, preferentially infiltrating non-lymphoid tissues to exacerbate local inflammation and tissue degeneration [90]. Importantly, T cell senescence can be pathologically accelerated—coinfection with SARS-CoV-2 and CMV potently drives premature immunosenescence via inflammatory microenvironment induction and exhaustion mechanisms, thereby elevating risks for cardiovascular and other chronic conditions [91].

### B-cell and immunosenescence

Immunosenescence exerts multifaceted effects on immune function, with particularly complex consequences for B cells [92]. The hallmarks of B cell immunosenescence include dysregulated cytokine secretion, aberrant immunoglobulin production, and impaired immunomodulatory capacity [93]. With advancing age, senescence is accompanied by adipose tissue expansion and elevated palmitic acid (FA) levels. Obesity exacerbates B cell immunosenescence through dual mechanisms involving leptin (an inflammatory driver) and FA (metabolic reprogramming). Increased adipocyte-derived leptin acts directly on B cells, upregulating mRNA expression of proinflammatory cytokines (TNF-α, IL-6, IL-8), chemokines (e.g., CXCL13), pattern recognition receptor TLR4, and the cell cycle inhibitor p16, thereby promoting a proinflammatory B cell phenotype. Additionally, leptin-treated B cells exhibit reduced class-switching capacity (IgM→IgG) and compromised regulatory function. Concurrently, FA enters B cells via metabolic pathways, inducing T-bet (a Th1-associated transcription factor) expression and stimulating autoreactive IgG antibody secretion. These changes accelerate the shift toward a proinflammatory/autoreactive B cell phenotype, leading to age-associated functional defects such as diminished proliferative capacity and impaired immune response efficiency [92, 94, 95]. Immunosenescence further modulates B cell trafficking under inflammatory conditions. Age-dependent elevation in inflammatory mediators alters homeostatic B cell migration into the peritoneal cavity, exacerbating inflammation-related pathologies [96]. This process heightens systemic inflammation, increasing vulnerability to infections and impairing vaccine responsiveness [92]. For instance, elderly individuals exhibited weaker antibody responses to influenza vaccine strains, though elevated BTLA levels on mature B cells enhances IgG reactivity against H1N1. Heightened BTLA levels on class-switched memory B lymphocytes also correlate with maintained neutralizing antibody levels against viral pathogens and enhanced anamnestic immune reactivity following booster immunization [97]. While age-related immune decline predominantly affects adaptive immunity [98], IL-15—a pleiotropic cytokine—offers therapeutic potential. IL-15 monoclonal antibody (mAb) treatment reduces the frequency of age-related B cells (ABCs; B220 + CD11c + T-bet+) and germinal center (GC) B cells, ameliorating manifestations of age-related autoimmunity [99].

## Impact of immunosenescence on age-related diseases

**Fig. 4:**
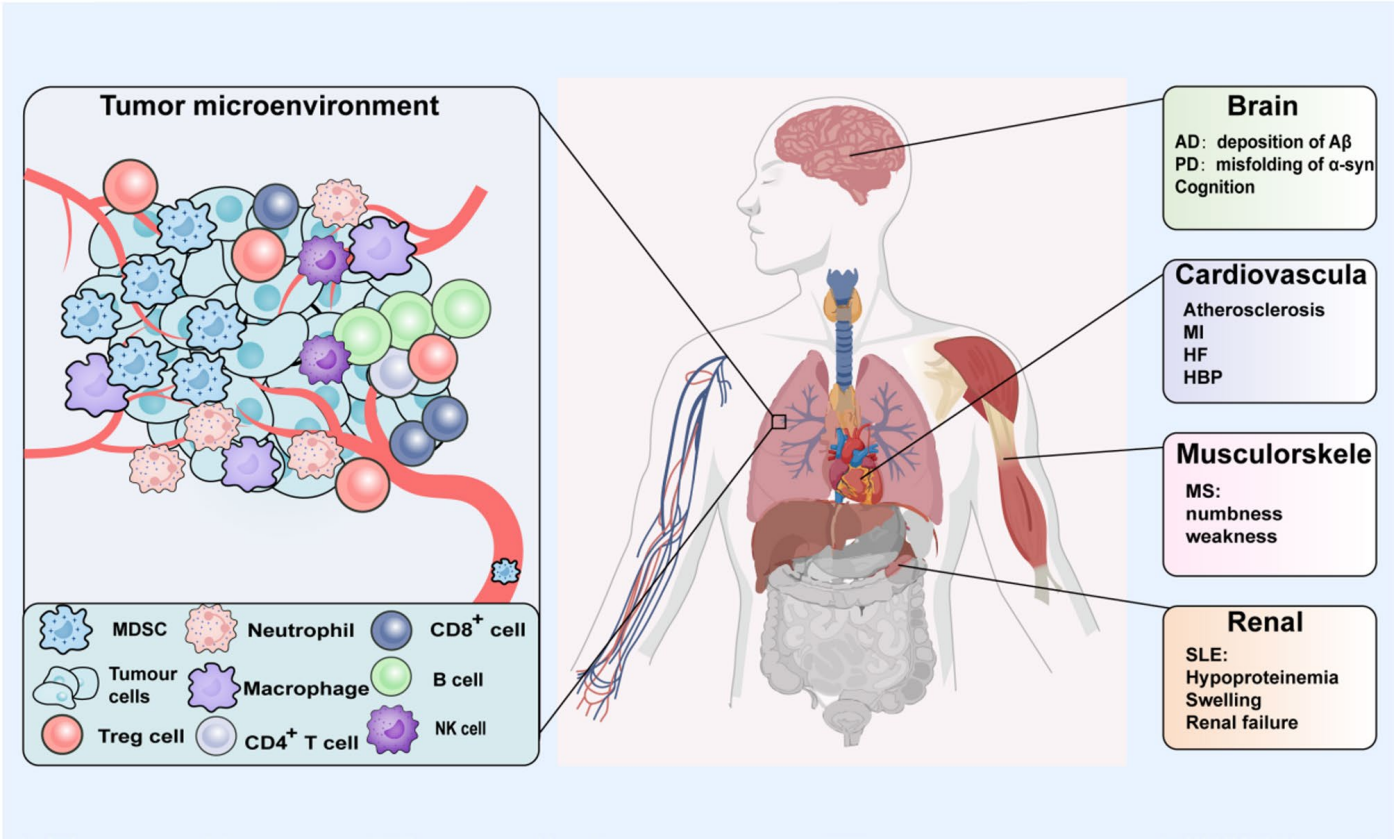
Schematic representation of immunosenescence-related diseases. Immunosenescence contributes to age-related immune dysfunction and is implicated in the onset and progression of various age-associated diseases through multiple mechanisms, including neurodegenerative disorders, cardiovascular diseases, malignancies, and autoimmune conditions.AD, Alzheimer’s disease; PD, Parkinson’s disease; MI, myocardial infarction; HF, heart failure; HBP, hypertension; SLE, systemic lupus erythematosus

With advancing age, immunosenescence emerges as a critical physiological phenomenon that not only compromises immune defense mechanisms but also significantly promotes the pathogenesis of multiple aging-associated disorders. Investigating the intrinsic connections and underlying mechanisms between immunosenescence and these disorders is essential for designing targeted interventions and promoting healthy longevity in the elderly(Fig. 4).

### Neurodegenerative diseases

Neurodegenerative diseases (NDs) are age-associated progressive neurological diseases featured by the gradual deterioration of cognitive, emotional, and motor functions in patients [86]. The pathological basis involves cellular senescence within the nervous system, particularly the accumulation and increased senescence of glial cells [100]. Representative disorders include Alzheimer’s disease (AD) and Parkinson’s disease (PD) [101, 102].

In AD, immunosenescence exacerbates neuroinflammation and neuronal damage through multiple mechanisms. First, age-related immune dysregulation disrupts microbial homeostasis, such as the excessive proliferation of oral anaerobic bacteria, which activates innate immune responses and elevates inflammatory mediators (e.g., TNF-α). This weakens the blood-brain barrier (BBB), establishing a detrimental inflammation-microbiome cycle that accelerates AD progression [103]. Second, immunosenescence impairs immune surveillance against latent pathogens like herpesviruses [104, 105], whose reactivation induces chronic inflammation and promotes AD pathogenesis [106]. Zoonotic pathogens such as Mycobacterium avium subspecies Paratuberculosis (MAP) further disrupt cellular metabolism and autophagy under immunosenescent conditions, leading to Aβ and tau accumulation and exacerbating neurodegeneration [107]. Additionally, senescent immune cells contribute to oxidative stress imbalance and enhanced lipid peroxidation, activating ferroptosis pathways [108, 109]. Dysregulation of the Nrf2-GPX4 axis impairs lipid peroxide clearance, triggering ferroptotic cascades that amplify neuronal damage in AD [110, 111]. Meanwhile, microglia in a pro-inflammatory state exhibit upregulated cytokine secretion and impaired Aβ clearance, forming a self-perpetuating cycle of neurodegeneration [112, 113]. Complement components (e.g., C3a) further increase BBB permeability and microglial activation, worsening neuroinflammatory responses [114].

PD pathogenesis is also closely linked to immunosenescence [115], which manifests through progressive deterioration of dopamine-producing neurons in the substantia nigra (SN) and aberrant α-synuclein (α-syn) aggregation, often accompanied by systemic chronic inflammation and immune dysregulation [116]. Immunosenescence disrupts T/B lymphocyte homeostasis, driving gut microbiota dysbiosis with pathogenic bacterial overgrowth and depletion of commensal species. This elevates circulating pro-inflammatory metabolites (e.g., lipopolysaccharides), inducing systemic inflammation. Immunosenescence modulates PD via the gut-microbiome-brain axis through two primary mechanisms: (1) Gut microbiota (GM) influences brain-gut axis dynamics by regulating immune, neuroendocrine, and enteric nervous systems, directly impacting motor function and PD onset [117]; (2) Microbial toxins induce α-syn aggregation in the enteric nervous system (ENS), which propagates to the central nervous system (CNS) through the vagus nerve, initiating neurodegeneration [118, 119]. α-syn aggregates activate microglia, promoting NLRP3 inflammasome-dependent IL-1β release and amplifying neuroinflammation in PD [120]. Furthermore, IL-17 A disrupts the BBB, activates microglia, and enhances TNF-α secretion and T-cell infiltration, exacerbating neurodegeneration [121]. Enteric glial cells also contribute to PD pathogenesis, as Toll-like receptor-mediated immune activation compromises intestinal barrier integrity, representing another critical mechanism in PD development [122, 123].

### Cardiovascular diseases

Studies have confirmed that immunosenescence plays a critical role in the development of cardiovascular disorders (CVD) [124]. DNA viruses (e.g., cytomegalovirus, CMV) accelerate immunosenescence by promoting endothelial injury and fostering a pro-inflammatory vascular microenvironment, thereby exacerbating atherogenesis [125]. Conversely, RNA viruses (e.g., HCV, HIV, and SARS-CoV-2) induce oxidative stress, DNA damage, and SASP, further aggravating vascular inflammation and cellular senescence [126]. The decline in dehydroepiandrosterone (DHEA) concentrations that accompanies aging has been shown to negatively impact the functional capacity of macrophages, leading to uncontrolled overproduction of IL-6 and TNF-α. Notably, foam macrophages (CD14 + CD16+) and intermediate-phenotype macrophages (LB-foam macrophages) aberrantly secrete human endogenous retrovirus K102 (HERV-K102) particles, amplifying inflammatory responses via “trained innate immunity” mechanisms, which play a critical role in advanced atherosclerotic plaque formation [127]. Telomere attrition exerts a dual effect: it not only diminishes immune cell replicative capacity and impairs lymphocyte and macrophage function but also promotes the secretion of pro-inflammatory mediators, resulting in “dysfunctional yet hyperactive” senescent immune cells that perpetuate vascular inflammation [128]. Additionally, immunosenescence modulates atherosclerosis through SASP, where senescent immune cells continuously release inflammatory mediators (IL-1β, IL-18) and chemokines, recruiting more immune cells into the vascular wall and establishing a chronic inflammatory loop that disrupts vascular integrity, accelerates lipid deposition, and promotes plaque instability [126]. Furthermore, immunosenescence drives the expansion of pro-inflammatory immune subsets, including granzyme K (GzmK) + CD8 + T cells and integrin α11b (CD11b) + integrin α11c (CD11c) + T-bet + B cells (ABC cells). These ABC cells exhibit enhanced antigen presentation and co-stimulatory molecule expression, intensifying intraplaque immune responses and accelerating disease progression [129]. Age-related decline in endoplasmic reticulum (ER) chaperones and foldases compromises protein folding and the unfolded protein response (UPR), increasing susceptibility to CVD in the elderly [130]. These intertwined mechanisms collectively establish a “immunosenescence-inflammation-atherosclerosis” vicious cycle, ultimately leading to vascular dysfunction and plaque formation.Beyond vascular effects, aging reduces ventricular nerve density and disrupts vasculature-derived neuromodulatory genes, impairing cardiac function. Studies revealed that aging downregulated microRNA-145 (miR-145), reducing semaphorin-3 A (Sema3a) expression and diminishing cardiac axon density, thereby destabilizing electrical signaling [131]. Moreover, age-associated adipose tissue expansion exacerbated systemic inflammation via adipokine dysregulation, disrupting metabolic homeostasis and increasing myocardial infarction risk [130]. Additionally, the CD4/CD8 ratio rose with age, and this T-cell subset imbalance was linked to elevated hypertension risk [132].

### Malignant tumors

Malignant tumors are frequently associated with marked immunosuppression [133]. The immunoregulatory network within the TME critically modulates cancer progression through intercellular communication mechanisms [110]. During immunosenescence, senescent T cells exhibit diminished responsiveness to cognate antigens and remain in a terminally differentiated, proliferatively exhausted state, compromising their capacity to identify and eliminate malignant cells, thereby fostering an immunosuppressive TME. Early hypotheses posited immunosenescence as an adaptive regulatory mechanism that maintains homeostasis by preventing excessive immune activation. However, recent studies demonstrate that SASP secretion exacerbates oncogenesis. For instance, senescent macrophages enhanced the proliferative, invasive, and metastatic potential of lung cancer cells via inflammatory mediators (e.g., IL-6, MMPs, ROS) within the TME [134]. Furthermore, metabolic dysregulation in immune cells (T cells and macrophages) may drove TME remodeling, creating a permissive niche for cancerous cells growth in elderly patients [135]. Notably, age-dependent tumorigenesis correlates strongly with impaired immune surveillance and dysregulated cancer immunoediting [136]. Compared to younger patients, geriatric oncology patients (≥ 75 years) often faced poorer prognoses due to immunocompromised status, higher comorbidity burdens, and reduced tolerance to treatment-related toxicity [137].

### Autoimmune diseases

The deterioration of immune function with aging exhibits a significant correlation with autoimmune diseases (AIDs), as it often coincides with elevated inflammatory levels, these mechanisms collectively represent major pathogenic factors in aging-associated disorders [138]. The progression of autoimmune diseases may be influenced by immunosenescence through multiple mechanisms. For instance, with increasing age, the generative ability of T cells markedly diminished, leading to an insufficient immune response to foreign pathogens.Immunosenescence disrupts immune tolerance and activates autoreactive T cells by reducing TCR diversity, impairing regulatory T-cell (Treg) function, and promoting the release of senescence-associated inflammatory factors. This perpetuates a vicious cycle of chronic inflammation and tissue antigen exposure, ultimately driving AIDs pathogenesis. Notably, TCR repertoire analysis has been employed to explore the influence of aging on multiple sclerosis (MS) [139]. Furthermore, immunosenescence affects autoimmune disease progression through age-related B cells (ABCs). These cells acquire a proinflammatory phenotype due to senescence-related dysfunction (e.g., mitochondrial abnormalities, epigenetic dysregulation), secreting IL-6 and TNF-α while producing autoreactive antibodies, thereby disrupting immune tolerance. Additionally, ABCs exacerbate chronic inflammatory microenvironments by activating senescence-related signaling pathways (e.g., mTOR/NF-κB), promoting abnormal activation and tissue infiltration of autoimmune cells (e.g., Th17), ultimately accelerating the pathological progression of rheumatoid arthritis (RA), systemic lupus erythematosus (SLE), and related disorders [140].

Immunosenescence-based therapies are emerging as novel treatment strategies for AIDs. In SLE, accelerated senescence of immune cells is accompanied by elevated IL-15 levels, leading to systemic immune hyperactivation. Studies demonstrated that targeting IL-15 or selectively eliminating senescent immune cells effectively reduced anti-dsDNA antibody titers and ameliorated renal damage, offering a promising therapeutic avenue [99]. Similarly, immunosenescence is implicated in disease progression, treatment response, and prognosis in MS. Recent research has explored innovative approaches, such as senolytic agents, neuroprotective interventions, and remyelination therapies, which may benefit elderly MS patients [141]. Concurrently, soluble costimulatory molecules (e.g., sCD163, sCD28, sCD80, sCTLA-4) have been proposed as promising indicators for assessing immunosenescence, facilitating the timely identification of AIDs and therapeutic response evaluation [142].

## Immunosenescence and histone post-translational modifications

**Fig. 5:**
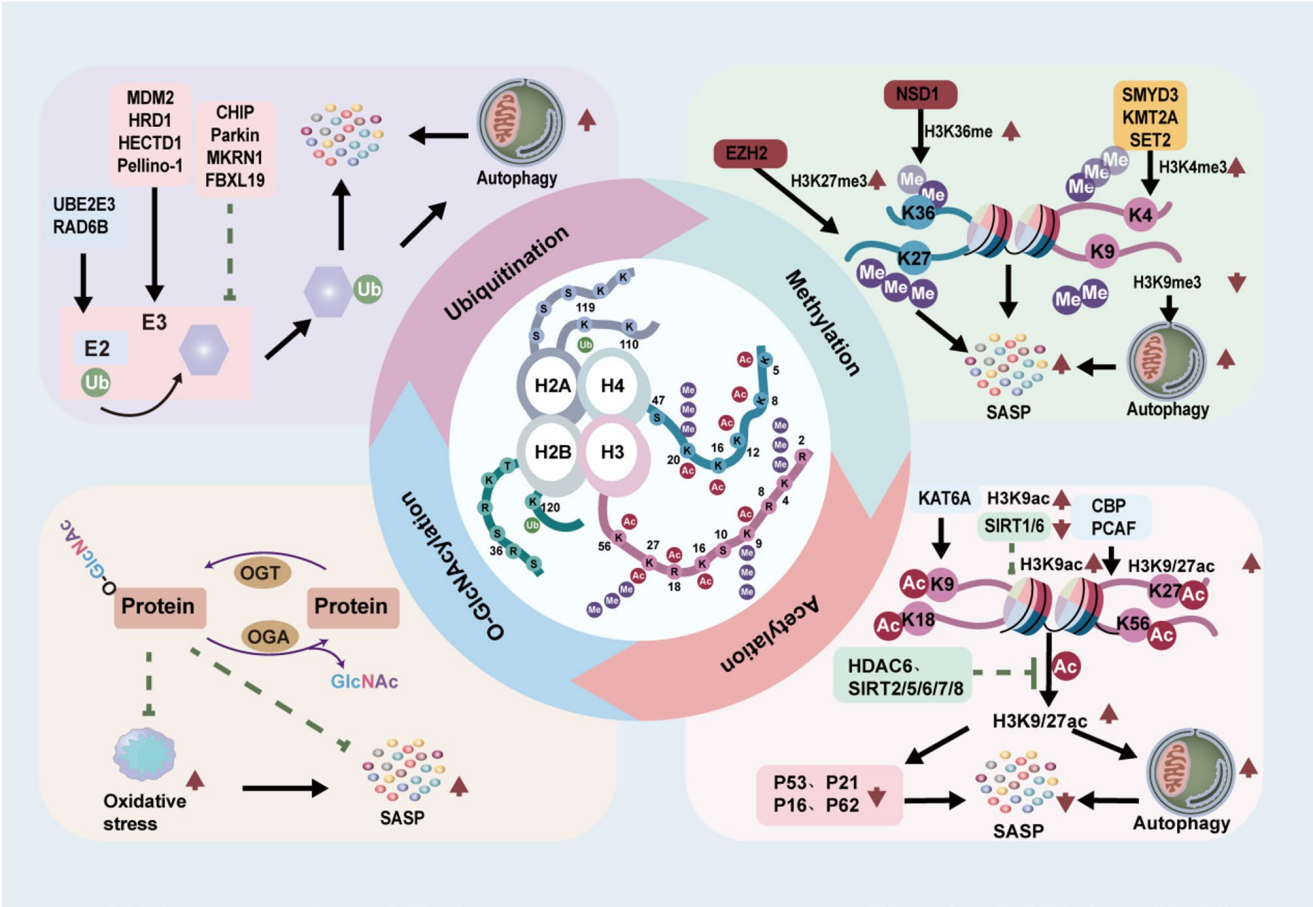
Histone modifications in immunosenescence. The four core histones—H2A, H2B, H3, and H4—undergo various post-translational modifications, including methylation, acetylation, ubiquitination, and O-GlcNAcylation at specific amino acid residues. These modifications play critical roles in the regulation of immunosenescence and the development of related diseases

PTMs serve as critical epigenetic regulators of cellular function during aging. In eukaryotic cells, numerous essential proteins - particularly those governing gene expression, cell cycle progression, DNA repair mechanisms, and metabolic regulation - undergo diverse PTMs. The eukaryotic genome is compacted into chromatin, with nucleosomes constituting its fundamental repeating units. Each nucleosomal core particle contains 146 bp of DNA wrapped around an octameric histone core comprising H3, H4, H2A, and H2B [143]. Specialized histone-modifying enzymes catalyze various PTMs at specific amino acid residues (lysine, serine, and arginine) within the flexible N-terminal tails of histones. These modifications encompass a broad spectrum of chemical alterations, including acetylation, methylation, phosphorylation, citrullination, ubiquitination, ADP-ribosylation, deamidation, formylation, O-linked β-N-acetylglucosamine modification (O-GlcNAcylation), as well as various acylations (propionylation, butyrylation, crotonylation) and proline isomerization [144, 145]. This section focuses on four major histone modifications (methylation, acetylation, ubiquitination, O-GlcNAcylation and PARylation(Fig. 5)) and reviews recent advances in understanding their implications in immunosenescence**(**Tables 1 and 2**).**

### Methylation

Methylation represents a pivotal form of PTMs, extensively occurring in various intracellular proteins, particularly in chromatin-associated histones and non-histone molecules. Among these, histone methylation stands out as one of the most stable and long-lasting PTMs, serving as an extensively studied mechanism for transcriptional inhibition and genetic regulation [146]. However, depending on the specific amino acid modified, histone methylation can also facilitate transcriptional activation [147]. This modification involves the addition of one or more methyl (–CH₃) groups to amino acid residues, primarily lysine (K) or arginine (R) [148]. Lysine residues can undergo mono- (me1), di- (me2), or trimethylation (me3) at their ε-amino groups, whereas arginine methylation may occur as monomethylation (me), symmetric dimethylation (me2s), or asymmetric dimethylation (me2a) [149]. The methylation process is predominantly catalyzed by histone methyltransferases (HMTs), which are divided into two major classes depending on their target amino acids: histone lysine methyltransferases (KMTs) and protein arginine methyltransferases (PRMTs). The KMT family includes enzymes such as EZH2, G9a, DOT1L, and SETD2, while the PRMT family in mammals comprises PRMT1 through PRMT9 [150]. Histone H3 serves as the primary site for methylation, though other core histones also undergo this modification [151]. Specifically, five lysine residues on histone H3 (K4, K9, K27, K36, and K79) are susceptible to methylation, whereas histone H4 is predominantly methylated at K20 by specific KMTs. Notably, methylation at H3K4 and H3K36 is associated with transcriptional activation, whereas methylation at H3K9, H3K27, H3K79, and H4K20 correlates with transcriptional repression [11].

Emerging research has increasingly linked histone methylation to immunosenescence and age-associated diseases. Emerging research indicated that site-specific histone methylation profoundly influences aging by modulating gene expression, autophagy, and inflammatory cytokine release. For instance, H3K4 methylation generally exhibited pro-aging effects. In intervertebral disc degeneration (IVDD) models, KMT2A-mediated H3K4me3 upregulated methyltransferase-like 3 (METTL3), leading to reduced ATG4a mRNA stability via m6A modification, thereby suppressing autophagy in nucleus pulposus cells (NPCs) and accelerating SASP expression [152, 153]. Conversely, in dietary-restricted (DR) C. elegans models, limited S-adenosylmethionine (SAM) synthesis inhibited SET-2 (a homolog of human KMT2), thereby reducing H3K4me3 levels and subsequently activating TFEB/HLH-30 and PHA-4/FOXA pathways to enhance autophagy and extend lifespan [154]. Similarly, in angiotensin II (Ang II)-induced endothelial senescence, the histone methyltransferase Smyd3 promoted H3K4me3 deposition at the p21 promoter, enhancing its transcription and accelerating cellular aging [155]. In contrast, H3K9me3 modification is predominantly localized to heterochromatin, and its abundance declines with advancing age [156]. Heterochromatin plays a pivotal role in transcriptional silencing and the maintenance of genomic stability [157]. The widespread loss of heterochromatin, especially constitutive heterochromatin, has been consistently recognized as a defining feature of aging across various cell types and species. This epigenetic alteration is primarily characterized by the depletion of H3K9me3, leading to loss of gene silencing and driving senescence-associated pathways [158]. Studies in progeria-derived fibroblasts (PSFs) demonstrated that heterochromatin reduction, marked by diminished H3K9me3 levels, suppressed autophagic flux, exacerbated SASP secretion, and subsequently induced cardiac fibrosis [159]. In a murine myocardial infarction (MI) model, loss of H3K9me3 sensitized mesenchymal stem cells (MSCs) to stress-induced apoptosis and premature senescence [160]. H3K27 methylation also represents a pivotal epigenetic regulator of cellular senescence and organismal aging. The Polycomb repressive complex 2 (PRC2), composed of EZH2, EED, and SUZ12, exclusively catalyzes H3K27me3, with PRC2 binding further activating EZH2 [161]. In human diploid fibroblasts (HDFs), EZH2 downregulation triggered DNA damage responses, subsequently activating p16 and SASP to induce senescence, establishing H3K27me3 as a senescence marker [162, 163]. Recent studies have demonstrated that both EZH2 and its associated histone modification marker, H3K27me3, exhibit age-dependent upregulation in the gastric tissues of aged mice, including both naturally aged and Klotho-deficient models [164]. Additionally, in mechanically induced osteoarthritis, miR-350-3p suppressed the H3K36 methyltransferase NSD1, reducing H3K36me levels and promoting chondrocyte senescence [165]. Arginine methylation also contributes to aging. PRMT1, the most prominent PRMT, regulated STAT1, lymphocyte signaling, and TNFα/NF-κB pathways, implicating it in immune responses [166]. PRMT1 deficiency induced mitochondrial dysfunction, exacerbating age-related sarcopenia [167]. In COPD models, PRMT1 depletion increased cellular senescence and death, worsening disease progression [168].

Lysine demethylases (KDMs), which utilize iron and 2-oxoglutarate (2OG) to oxidatively remove methyl groups, play pivotal roles in gene regulation and chromatin structure [169, 170]. Among these, KDM1A and KDM2A are key regulators of aging. KDM1A safeguarded PRC2-mediated gene silencing boundaries, preventing age-associated euchromatinization in neurons [171]. KDM2A depletion elevated H3K36me2, which recruited DNA repair factors and enhanced genomic stability, thereby delaying senescence [172]. Conversely, KDM3A and KDM4C regulated heterochromatin reorganization in mesenchymal stromal cells (MSCs) by activating condensins NCAPD2 and NCAPG2. Their inhibition induced DNA damage responses, exacerbating cellular aging [173]. KDM6A, however, exerted detrimental effects by demethylating H3K27me3/H3K4me3, promoting IRF5 transcription and X-chromosome escape, thereby amplifying pro-inflammatory microglial responses in aging [174]. Additionally, KDM6A interacted with calponin 1 (CNN1) to form an epigenetic axis that regulated trauma-induced senescence in spinal cord microvascular endothelial cells (SCMECs), influencing neuroinflammation and functional recovery [175].

### Acetylation

The balance of histone acetylation is maintained by two counteracting enzymes: HATs and HDACs, which maintain a delicate equilibrium to ensure precise gene expression control [12]. HATs catalyze the transfer of acetyl groups to lysine residues on histone tails and are phylogenetically classified into two major families: the Gcn5-related N-acetyltransferases (GNAT) and the MYST family [176, 177]. The GNAT family, comprising Gcn5, p300/CBP-associated factor (PCAF), Elp3, and Hpa2, primarily mediates acetylation at lysine sites of histone H3 through an ordered sequential mechanism involving direct nucleophilic attack of the ε-amino group on enzyme-bound acetyl-CoA. In contrast, the MYST family—including catalytic subunits such as Moz, Ybf2/Sas3, Sas2, Tip60, MORF, and HBO1—preferentially acetylates histone H4 lysine residues via a direct attack mechanism within the Esa1×acetyl-CoA×histone ternary complex. Notably, certain HATs also post-translationally modify non-histone proteins, including high-mobility group chromatin proteins, transcriptional activators (e.g., p53), coactivators, and general transcription factors [177, 178]. The reverse reaction, involving acetyl group removal from histone tails, is catalyzed by HDACs. Mammalian HDACs are currently categorized into four classes: Class I (HDAC1-3, HDAC8), Class II (HDAC4-7, HDAC9-10), Class III (SIRT1-7), and Class IV (HDAC11). Mechanistically, Class I, II, and IV HDACs are zinc-dependent metalloenzymes, whereas Class III HDACs (sirtuins) require NAD + as an essential cofactor for their deacetylase activity [179].

HATs are primarily regulated by acetyl-CoA levels. In tissues, fasting or caloric restriction reduces cytoplasmic acetyl-CoA, leading to diminished p300 HAT activity, which in turn promotes longevity-associated autophagy. Conversely, elevated nuclear acetyl-CoA enhances HAT activity and increases histone acetylation, paradoxically extending lifespan. Additionally, The nuclear translocation of cytoplasmic acetyl-CoA synthetase 2 (ACSS2) enhances the activity of HATs, including CREB-binding protein (CBP), p300/CBP-associated factor (PCAF), and others. This activation increases H3K9 and H3K27 acetylation at neuronal gene promoter regions. Moreover, ACSS2 interacts with transcription factor EB (TFEB) to facilitate H3K9 acetylation at TFEB target gene promoters, thereby promoting lysosomal biogenesis and autophagy. These processes collectively counteract age-related cellular decline [180]. Among the lysine acetyltransferases (KATs) of the MYST family (KAT5-KAT8), KAT6A suppresses cellular senescence by modulating repressors at the CDKN2A locus via H3K9 acetylation [181]. Elongator complex subunit 3 (Elp3), the catalytic component of the elongator complex, facilitates H3 acetylation of α-tubulin, which functions as a molecular beacon for motor protein attachment to microtubules. This process influences neuronal protein and organelle transport, exacerbating neurodegeneration in aging-related disorders. Furthermore, α-tubulin H3 acetylation is negatively regulated by HDAC6 and SIRT2/5/6/7/8 [182]. During aging, depletion of the mediator complex subunit Med5 or mutations in Med7 lead to its reduced occupancy near subtelomeric X-elements, disrupting the balance between silent information regulator 2 (Sir2) and silence-associated sequence 2 (Sas2). The relative increase in Sas2 activity elevates H4K16 acetylation near telomeres, inducing subtelomeric gene silencing. This epigenetic alteration aligns with the observed increase in telomeric H4K16 acetylation in senescent cells, revealing Sas2’s role in aging via modulation of telomeric histone acetylation [183]. Under persistent DNA damage, nuclear DNA damage signaling activates mitochondrial β-oxidation, triggering adipose tissue breakdown and generating excess acetyl-CoA to fuel histone acetylation. Tip60, a central metabolic-epigenetic regulator, not only facilitates polyunsaturated fatty acid accumulation and lipid mediator production but also directly catalyzes H4 hyperacetylation, collectively reshaping chromatin architecture. This process alters the expression profiles of immune effectors and cytochrome genes, establishing a pro-inflammatory microenvironment resembling immune activation, ultimately driving age-related functional decline [184]. In aged hematopoietic stem cells (HSCs), a marked reduction in H4K77ac acetylation had been observed, which was associated with enhanced HDAC3 activity. Notably, inhibition or knockdown of HDAC3 in HSCs significantly promoted lymphoid lineage differentiation [185]. Moreover, histone acetylation profiling of the choroid in aged mice revealed a distinct molecular signature characterized by decreased levels of H3K14ac, H3K56ac, and H4K16ac [186].

Sirtuins, classified as class III HDACs, serve as critical sentinels for maintaining genomic stability by mediating lysine residue deacetylation in an NAD⁺-dependent manner. SIRT1 was downregulated by lysosomal activity in hematopoietic and immune organs following natural aging of T cells in mice and elderly humans [187]. In SIRT1-deficient cells, SASP components, including IL-6 and IL-8, rapidly accumulated. Mechanistically, SIRT1 bound to the promoter regions of IL-8 and IL-6 but dissociated during cellular senescence, coinciding with progressive hyperacetylation of histone H3K9 and H4K16 at these loci [188]. SIRT6 exerted pleiotropic protective effects in endothelial cells by deacetylating histone H3K9, thereby enhancing endothelium-dependent vasodilation, promoting vascular NO bioavailability, decreasing membrane permeability, attenuating endothelial senescence and apoptosis, and stimulating autophagy [189]. In aged mice, SIRT2 deficiency led to superoxide and ROS accumulation, exacerbating age-related arterial stiffness and impaired contractile relaxation, suggesting that SIRT2-mediated deacetylation served as an adaptive response to delay vascular aging [190]. Furthermore, HDAC1/2-deficient podocytes in mice exhibited hallmark senescence features, including elevated age-associated β-galactosidase (SA-β-gal) activity and lipofuscin accumulation [191]. Intriguingly, HDAC1 can be ubiquitinated and degraded by murine double minute 2 (MDM2), thereby promoting senescence [192]. In COPD, lymphocyte senescence was linked to HDAC2 loss in CD28-null CD8⁺ T cells and NKT-like cells [193]. Additionally, HDAC3-deficient bone marrow stromal cells (BMSCs) displayed chromatin alterations associated with premature senescence and lipid droplet (LD) formation, indicating that HDAC3 is crucial in suppressing skeletal aging by regulating LD biogenesis and senescence in osteochondroprogenitor cells [194].

### Ubiquitination

Ubiquitin (Ub), an evolutionarily conserved 76-amino acid regulatory polypeptide, was initially characterized by Goldstein and colleagues in 1975 [195]. Ubiquitination involves the covalent conjugation of Ub to the ε-amino group of lysine residues on substrate proteins, a process mediated by the sequential action of E1 activating enzymes, E2 conjugating enzymes, and E3 ubiquitin ligases [196]. Ub itself contains seven lysine residues (K6, K11, K27, K29, K33, K48, and K63) and an N-terminal amine, enabling the formation of polyubiquitin chains through linkage at these sites—a phenomenon termed polyubiquitination. Conversely, deubiquitinating enzymes (DUBs) catalyze the removal of ubiquitination from substrates [197].

During aging, ubiquitination modifications extensively participate in cell fate determination and tissue functional decline through diverse mechanisms, including proteostasis regulation, mitochondrial function, oxidative stress, and inflammatory responses. E3 ubiquitin ligases serve as pivotal regulators of senescence by facilitating the ubiquitin-dependent degradation of specific substrates. For instance, the activity of the CHIP ligase was modulated by the DPP4-MYH9 axis, wherein DPP4 competitively bound MYH9 to suppress CHIP function, thereby exacerbating oxidative stress and senescence in chondrocytes [13]. In nucleus pulposus cells, secreted phosphoprotein 1 (SPP1) impeded PINK1/Parkin-mediated mitophagy via the integrin α5β1 pathway, accelerating cellular senescence and intervertebral disc degeneration [198]. Furthermore, studies in diabetic nephropathy (DN) mouse models demonstrated that the E3 ligase Parkin mitigated renal tubular epithelial cell (RTEC) senescence by alleviating inflammation and fibrosis [14]. Similarly, FBXL19 exerted cardioprotective effects during cardiac aging by enhancing innate immune responses and suppressing inflammation and cellular senescence [15]. Atherosclerosis research further revealed a close association between E3 ligases and the SASP. MKRN1 deficiency activated the NF-κB pathway in endothelial cells, aggravating endothelial dysfunction and inflammation [199].In the nervous system, cadmium chloride exposure activated the SEL1L/HRD1-mediated ERAD system, leading to SigmaR1 ubiquitination and accelerated neuronal senescence [200]. Analogously, HECTD1 mediated 4-hydroxynonenal (4HNE)-induced senescence in RAW264.7 cells by targeting IRS1 for degradation, a process counteracted by the soluble anti-aging factor α-Klotho [201]. In COPD models, Pellino-1 modulated p21-dependent SASP production, and its silencing attenuated chronic inflammation and tissue aging [202]. Additionally, MDM2 exacerbated renal aging by ubiquitinating HDAC1 and promoting its degradation [192]. E2 ubiquitin-conjugating enzymes also play critical roles in senescence. For example, miR-143-3p restricted Sertoli cell proliferation and disrupted the blood-testis barrier by targeting the ubiquitin-conjugating enzyme UBE2E3 [203]. RAD6B deficiency accelerated retinal aging, resulting in increased apoptosis and DNA damage [204].

DUBs influence aging by modulating the ubiquitination status of specific proteins. In premature ovarian insufficiency (POI), USP14 promoted DNA damage accumulation, hastening ovarian aging [205]. USP11 enhanced renal senescence and fibrosis by deubiquitinating and stabilizing TGF-β type II receptor (Tgfbr2), thereby amplifying senescence-associated signaling [206]. USP13 contributed to pulmonary aging by regulating K63-linked ubiquitination of MDM2, inducing cellular senescence [207]. Conversely, USP18 alleviated inflammation and senescence in coronary endothelial cells [208], while USP30 silencing ameliorated mitochondrial dysfunction and cardiomyocyte senescence [209]. Notably, USP3 exerted protective effects in age-related disorders by inhibiting chondrocyte senescence via SIRT3 and FOXO3 deacetylation [210].

### O-GlcNAcylation

O-GlcNAcylation represents a non-canonical glycosylation modification marked by the covalent conjugation of a single O-linked N-acetylglucosamine (O-GlcNAc) moiety to serine and threonine residues of cytosolic, nuclear, and mitochondrial proteins, constituting a dynamic and reversible PTMs [211]. This modification is catalyzed by OGT, while its removal is mediated by O-GlcNAcase (OGA). Together, these enzymes maintain the dynamic equilibrium of O-GlcNAc levels in response to cellular metabolic states and environmental fluctuations.

Recent studies underscored the critical involvement of O-GlcNAcylation in aging and associated pathologies, particularly in neurodegenerative disorders and metabolic homeostasis [212, 213]. In mammals and the model organism Caenorhabditis elegans, O-GlcNAcylation directly modulated key longevity-associated transcription factors, such as Nrf-2 (SKN-1 in C. elegans), promoting their nuclear accumulation and enhancing antioxidant defense mechanisms, thereby extending lifespan [16]. O-GlcNAcylation had also been demonstrated to be essential for synaptic plasticity and cognitive function. Studies revealed that aged mice exhibited reduced OGT expression and altered O-GlcNAcylation patterns in the brain, which correlated with cognitive decline. Notably, OGT overexpression in the hippocampus of aged mice partially restored spatial learning and memory, underscoring the critical role of O-GlcNAc regulation in maintaining neuroplasticity and cognitive function [214].The engagement of O-GlcNAcylation in NDs is particularly significant. Enhanced O-GlcNAcylation mitigates the phosphorylation and aggregation of tau and β-amyloid in AD, alleviating neurotoxicity [17, 215]. Intriguingly, ischemia-induced O-GlcNAcylation was compromised in the aged brain, whereas augmenting O-GlcNAcylation improved functional recovery after ischemic injury [216]. Furthermore, O-GlcNAcylation modulated antiviral defense pathways. Recent findings indicated that O-GlcNAcylation suppressed senescence-induced β-hydroxybutyrylation (Kbhb) at Thr699 of STAT1, thereby strengthening the interaction between STAT1 and interferon-α/β receptor 2 (IFNAR2) and enhancing IFN-I-mediated antiviral responses. This mechanism offered a potential therapeutic strategy to bolster antiviral defenses in the elderly [217].

### PARylation

Poly ADP-ribosylation (PARylation) is a post-translational modification catalyzed by poly(ADP-ribose) polymerases (PARPs), in which ADP-ribose units derived from NAD⁺ are covalently attached to target proteins as linear or branched polymers, thereby modulating their structure and function. PARP1, the most extensively studied member of the PARP family, is rapidly activated in response to DNA damage and facilitates the recruitment of repair proteins through auto-PARylation [218]. Since its discovery, accumulating evidence has demonstrated that PARylation plays a critical role in cellular stress responses and DNA repair, closely associated with repair efficiency and fidelity.Beyond its canonical role in genome maintenance, PARylation has emerged as a pleiotropic regulator implicated in inflammation, metabolism, and cell death, highlighting its dual impact on cellular homeostasis. Under physiological conditions, PARylation contributes to immune defense by promoting the release of pro-inflammatory cytokines such as TNF-α and IL-6 in macrophages. However, during aging, its excessive activation can trigger inflammaging, thereby accelerating immunosenescence. Chronic inflammation, in turn, has been linked to telomere shortening and functional decline in T cells, as well as an increased risk of cardiovascular disease [219, 220].Recent studies have further revealed the multifaceted involvement of PARylation in the progression of immunosenescence. For example, PARylation has been shown to regulate the activation of both T and B lymphocytes [219]. In senescent T cells, aberrant PARP1 activity disrupted the modification balance of LAT, a critical adaptor in TCR signaling, thereby impairing T cell proliferation and differentiation. Similarly, in aged B cells, dysregulated expression of PARylation-related factors reduced their ability to produce high-affinity antibodies.Moreover, PARylation also modulated macrophage metabolic polarization. In aging, abnormal PARP activity enhanced glycolysis in pro-inflammatory M1 macrophages and suppressed the anti-inflammatory functions of M2 macrophages [220]. Notably, PARylation was implicated in the regulation of DNA methylation patterns and metabolic homeostasis, with its overactivation associated with disrupted glucose metabolism and the development of type 2 diabetes [221].Taken together, PARylation contributed to immunosenescence through multiple dimensions, including immune cell activation, metabolic reprogramming, and epigenetic regulation, offering new mechanistic insights and potential therapeutic avenues for delaying immune aging and preventing age-related diseases.

## Therapeutic strategies targeting post-translational modifications in Immunosenescence

During immunosenescence, PTMs—including histone methylation, acetylation, ubiquitination, and O-GlcNAcylation—serve as pivotal regulators that influence both modulating gene expression and proteostasis, while also promoting pathological processes such as inflammatory activation and cellular functional decline. Targeting these modifications presents a promising avenue for anti-aging interventions(Table 3).

Histone methylation serves as a central epigenetic regulator with substantial therapeutic potential. High-intensity interval training (HIIT) has been demonstrated to downregulate KMT2D and its target gene IDI1, thereby suppressing H3K4me1-mediated activation of lipid metabolism-related promoters and disrupting the vicious cycle between metabolic dysregulation and chronic inflammation [222]. Notably, senolytic drug combinations (e.g., dasatinib + quercetin) modulate the H3K9me3/H3K27me3 landscape, attenuating systemic inflammation and improving cognitive function in aging models [223]. The EZH2 inhibitor EPZ6438 (Tazemetostat) has been shown to alleviate age-related decline of interstitial cells of Cajal (ICCs) in the mouse stomach [224]. Additionally, PRMT1-catalyzed H4R3me2a modification inhibits PA200-mediated H4 degradation. Intriguingly, anti-aging agents such as metformin, rapamycin, and resveratrol restore H4R3me2a levels, stabilize nucleosome architecture, and regulate SASP-related and anti-apoptotic gene expression, offering novel strategies for epigenetic-based senescence intervention [225].

As a fundamental epigenetic mechanism, histone acetylation exhibits broad therapeutic effects in aging modulation. HDAC1/2 inhibitors (e.g., trichostatin(TSA) and suberoylanilide hydroxamic acid(SAHA)) restored acetylation at H3K9 and multiple H4 residues, counteracting ROS accumulation and inflammation caused by EC-SOD downregulation in aged pulmonary fibroblasts [226]. Moreover, TSA suppressed SASP gene activation by reducing H3K9/14 hyperacetylation at promoter regions, demonstrating anti-fibrotic efficacy in Duchenne muscular dystrophy(DMD) [227]. Honokiol modulated H3 acetylation and γ-H2AX, inhibiting NF-κB while upregulating IL-10, thereby conferring dual anti-inflammatory and neuroprotective benefits in neurodegenerative disorders [228]. Chitinase 1 (CHIT1) ameliorated cognitive deficits and neuroinflammation via HDAC3 inhibition [229]. Valproic acid (VPA) mitigated diabetes-associated cellular senescence and SASP by suppressing C5a receptor expression, expanding the clinical applicability of HDAC inhibitors [230]. Furthermore, class III HDACs (e.g., SIRT1/SIRT6) regulated histone acetylation to counteract lymphocyte senescence in COPD and conferred vascular protection [189, 231].A clinical study demonstrated that time-restricted feeding (TRF) enhanced the expression of the deacetylase SIRT1 and promoted autophagy, thereby exerting anti-aging effects in humans [232]. Although nicotinamide, a class III HDAC inhibitor, was shown to improve cognitive function in Alzheimer’s disease (AD) mouse models, a phase 2a proof-of-concept clinical trial indicated that further development of nicotinamide as a potential intervention for AD must address challenges related to its limited bioavailability and rapid methylation metabolism [233].

As a critical component of the protein degradation system, ubiquitination modulated inflammatory signaling and aberrant protein clearance. In osteoarthritis (OA), DPP4 bound MYH9, impeding its CHIP-mediated ubiquitination and degradation, thereby exacerbating mitochondrial fission and SASP activation. The small-molecule 4,5-dicaffeoylquinic acid disrupted this interaction, restoring ubiquitin-proteasome function and alleviating cartilage degeneration [13]. In AD, the herbal compound “Bushen Huoxue Zhen” upregulated the E3 ligase MARCHF3, promoting NLRP3 ubiquitination and degradation, which suppressed pyroptosis and reduced IL-1β/IL-18 release, ultimately ameliorating neuroinflammation [234]. The natural triterpenoid Friedelin recruited RNF182 to facilitate K48-linked ubiquitination and autophagic clearance of p65, blocking NF-κB activation and attenuating tendinopathy-associated senescence [235].

O-GlcNAcylation, a nutrient and stress-responsive PTM, exerted dual roles in age-related neuropathology. For instance, the OGA inhibitor Thiamet-G enhanced O-GlcNAcylation in AD, reducing tau pathology and neuroinflammation [17]. Ischemia-induced O-GlcNAcylation impairment in the aging brain was linked to insufficient UDP-GlcNAc availability; however, Thiamet-G treatment improved neurofunctional outcomes following transient cerebral ischemia in both juvenile and senescent animal models [216].

## Conclusion and future perspectives

Epigenetic modifications play a multifaceted role in immune-inflammatory responses. Among these, histone modifications constitute a critical regulatory mechanism governing immune cell function during immunosenescence and age-related pathologies. With advancing research in this field, novel histone modifications closely associated with aging continue to be identified. Diverse experimental approaches have been employed to elucidate the functional implications of these modifications. Consequently, therapeutic strategies targeting histone-modifying enzymes have been developed and are currently under evaluation in both preclinical and clinical settings. Nevertheless, the current understanding of histone modifications remains incomplete, leaving numerous unresolved questions that warrant further investigation. Future therapeutic interventions aimed at modulating histone modifications to maintain immune homeostasis may hold significant clinical promise. In summary, deciphering the role of histone modifications in aging-associated immune inflammation is essential for unraveling the mechanisms of immunosenescence and developing novel treatment modalities.

**Table 1:** Histone methylation and acetylation during Immunosenescence

Potential Target	Modification Sites	Enzyme	Model	Mechanism	Effect	References
Methylation	H3K4me3	KMT2A	IVDD	KMT2A upregulates H3K4me3, promotes METTL3 expression, mediates m⁶A methylation, inhibits autophagy, and induces SASP	Promotes aging	[152, 153]
	H3K4me3	SET-2	Dietary restriction (DR) C. elegans model	Inhibits SET-2, reduces H3K4me3, promotes HLH-30/TFEB and PHA-4/FOXA, and promotes autophagy	Delays aging	[154]
	H3K4me2/3	Smyd3	Angiotensin II (Ang II)-induced rat endothelial cell aging model	Binds to the P21 promoter, promotes H3K4me3, and induces SASP	Promotes aging	[155]
	H3K9me3	–	Mouse myocardial infarction (MI) model	Lack of H3K9me3 promotes autophagy	Promotes aging	[156]
	H3K27me3	PRC2	Diploid fibroblast (HDF) aging model	After EZH2 downregulation, reduced H3K27me3 demethylation induces DNA damage, activates P16 and SASP	Promotes aging	[161]
	H3K27me3	EZH2	Naturally aging mice	Upregulation of H3K27me3	Promotes aging	[164]
	H3K36me	NSD1	Osteoarthritis mouse model	miR-350-3p inhibits NSD1, reducing H3K36me levels	Promotes aging	[165]
	–	PRMT1	PRMT1 knockout model	PRMT1 deficiency promotes cell death	Promotes aging	[166]
	H3K4me1/3	KDM1A	Kdm1a knockout model	KDM1A deficiency leads to downregulation of H3K4me1 and upregulation of H3K4me3, protecting the gene silencing boundary mediated by PRC and preventing age-related euchromatinization in neurons	Delays aging	[171]
	H3K36me2	KDM2A	SIRT6 knockout model	Deletion increases H3K36me2 levels, recruits DNA repair factors, and enhances genome stability	Delays aging	[172]
	H3K9me3	KDM3A/4 C	Mesenchymal stromal cell (MSCs) aging model	Downregulation of H3K9me3 activates condensins NCAPD2 and NCAPG2, regulates heterochromatin reorganization, and induces DNA damage	Promotes aging	[173]
	H3K27me3	KDM6A	KDM6A knockout model	KDM6A deficiency causes H3K27me3 accumulation and regulatory disorders, combined with X-chromosome escape, amplifying the pro-inflammatory response	Promotes aging	[174]
	H3K27me3	KDM6A	Spinal cord microvascular endothelial cell (SCMECs) aging model	Downregulation of H3K27me3 interacts with CNN1, affecting neuroinflammation and functional recovery	Promotes aging	[175]
Acetylation	H3K9/27ac	CBP, PCAF, and other HATs	–	Increases H3K9/K27ac in the promoter region of neuronal genes, binds to TFEB to promote H3K9ac in the promoter of target genes, and drives lysosomal biogenesis and autophagy	Delays aging	[177, 178]
	H3K9ac	KAT6A	Lymphoma mouse model	Upregulation of H3K9ac regulates the repressor at the CDKN2A locus	Delays aging	[181]
	H3ac	Elp3	–	Upregulation of H3ac affects neuronal protein and organelle transport, exacerbating neurodegeneration in aging-related diseases	Promotes aging	[182]
		HDAC6 and SIRT2/5/6/7/8	–	Downregulation of H3ac negatively regulates neuronal protein and organelle transport	Delays aging	[182]
	H4K16ac	Sir2/Sas2	Saccharomyces cerevisiae cell model	Imbalance of Sir2/Sas2, upregulation of H4K16ac, induces subtelomeric gene silencing	Promotes aging	[182]
	H4ac	Tip60	C. elegans model	Catalyzes H4 hyperacetylation, remodels chromatin structure, and establishes a pro-inflammatory microenvironment	Promotes aging	[184]
	H3K9/H4K16	SIRT1	Human fibroblast model	SIRT1 deficiency leads to upregulation of H3K9/H4K16ac, transcriptional activation and increased expression of SASP factors	Promotes aging	[188]
	H3K9	SIRT6	Hypertensive mouse model	SIRT6 deacetylates H3K9, regulates the Nkx3.2 - GATA5 pathway, protects endothelial function and resists hypertension	Delays aging	[189]
	K81	SIRT2	Sirt2 knockout mouse model	Sirt2 deficiency leads to downregulation of deacetylation of the aging regulatory protein p66Shc at K81, accumulation of superoxide and ROS, exacerbating age-related arterial stiffness and impaired contraction and relaxation	Delays aging	[190]
	–	HDAC1 /2	Nphs2-Cre Hdac1/2	HDAC1/2 deficiency leads to increased SA-β-gal activity and lipofuscin accumulation	Delays aging	[191]
	–	HDAC3	HDAC3 knockout mouse model	HDAC3 deficiency causes chromatin changes related to lipid droplet (LD) formation	Delays aging	[192]

**Table 2:** Histone ubiquitination and O-GlcNAcylation during Immunosenescence

Potential Target	Modification Sites	Enzyme	Model	Mechanism	Effect	References
Ubiquitination	K1642	CHIP	Mouse model of osteoarthritis (OA)	DPP4 competitively binds to the K1642 site of MYH9, blocking CHIP-mediated ubiquitin-proteasomal degradation, and the accumulation of MYH9 triggers excessive mitochondrial fission and exacerbates oxidative stress	Promotes aging	[13]
	-	Parkin	IVDD rat model	SPP1 inhibits mitophagy by blocking the PINK1/PARKIN pathway, accelerating nucleus pulposus cell degeneration and inducing calcification	Promotes aging	[198]
	–		Diabetic nephropathy (DN) mouse model	Parkin ubiquitinates GATA4, downregulating the GATA4/GAS1 signaling pathway and alleviating inflammation and fibrosis in renal tubular epithelial cells (RTEC)	Delays aging	[14]
	–	FBXL19	Cardiovascular injury model related to influenza A virus (IAV) infection	Enhances the innate immune response, inhibits inflammation and cell aging	Delays aging and protects the heart	[15]
	–	MKRN1	Endothelial cell (ECs) aging and activation model	High expression of MKRN1 induced by d-flow ubiquitinates and degrades related regulatory molecules, activates the NF - κB pathway and the cell aging program, and exacerbates endothelial cell dysfunction and promotes atherosclerosis	Promotes aging	[199]
	K142	HRD1	Neuron aging model exposed to cadmium chloride (CdCl₂)	Cadmium chloride exposure activates the SEL1L/ hrd1-mediated ERAD system, leading to ubiquitination of SigmaR1	Promotes aging	[200]
	–	HECTD1	RAW264.7 cell aging model	KL targets HECTD1, inhibits IRS1 ubiquitin-proteasomal degradation, regulates macrophage aging and function, and alleviates diabetic retinopathy	Promotes aging	[201]
	K63	Pellino-1	D-gal-induced mouse aging model	Pellino - 1 ubiquitinates p21 via the K63 site and participates in the aging process of lung cells and the development of COPD	Promotes aging	[202]
	–	MDM2	2-year naturally aging mouse model	MDM2 ubiquitination promotes HDAC1 degradation and promotes kidney aging	Promotes aging	[191]
	–	UBE2E3	Aging Sertoli cell (SCs) model	miR - 143 - 3p targets UBE2E3, restricting SCs proliferation and disrupting the blood-testis barrier	Promotes aging	[202]
	–	RAD6B	RAD6B knockout (KO) mouse model	RAD6B deficiency disrupts the PEDF signal, activates DNA damage, aging, and apoptosis pathways, and accelerates retinal degeneration	Delays aging	[204]
	K13/K15	USP14	D - gal-induced POI mouse model	Abnormally high expression of USP14 disrupts the DDR function of granulosa cells	Promotes ovarian aging	[205]
	–	USP11	Unilateral ureteral obstruction (UUO) mouse model	USP11 deubiquitinates Tgfbr2 to reduce its degradation, activating the downstream aging signaling pathway	Promotes kidney aging and fibrosis	[206]
	–	USP13	USP13 knockout mouse model	USP13 participates in the lung aging signaling pathway by regulating the K63-linked ubiquitination and protein stability of MDM2	Promotes lung aging	[207]
	–	USP18	Human coronary artery endothelial cells (HCAECs)	USP18 participates in the regulation of inflammation via the ubiquitination pathway	Delays aging	[208]
	–	USP30	D - gal-treated cell aging model	USP30 antagonizes Parkin and negatively regulates mitophagy, accelerating cardiomyocyte aging	Promotes aging	[209]
	–	USP3	Primary articular chondrocyte model	USP3 upregulates SIRT3 and inhibits IL - 1β-induced chondrocyte aging	Delays aging	[210]
O-GlcNAcylation	Ser470/Thr493	OGT	C. elegans model	OGT-1 can catalyze the O-GlcNAc glycosylation modification of the Ser470 and Thr493 sites of the SKN-1 protein, thereby affecting the accumulation, activity of SKN-1 in the intestinal nucleus, and the lifespan and oxidative stress resistance of nematodes	Delays aging	[16]
	–	OGT	OGT knockout mouse model	Decreased levels of OGT and O - GlcNAcylation in the brains of aging mice lead to cognitive impairment. Regulating OGT promotes cognitive rejuvenation	Delays aging	[214]
	–	OGA	O - GlcNAcylation overexpressing C57BL/6J mouse model	Inhibition of OGA affects AD treatment	Delays aging	[17, 215]
	–	OGT, OGA	Transient cerebral ischemia stroke model	The XBP1s/HBP/O - GlcNAc axis regulates neuroprotection in ischemic stroke	Delays aging	[216]
	Thr699	OGT, OGA	–	Modifies β - hydroxybutyrylation (Kbhb), enhances the interaction between STAT1 and IFNAR2, and enhances the antiviral response mediated by ifn - i	Delays aging	[217]

**Table 3:** Potential treatments targeting histone modifcations in Immunosenescence

Potential Target	Mechanism	Therapeutic Strategy	Efficacy	References
Methylation	Inhibits KMT2D and reduces H3K4me1	High - intensity interval training (HIIT)	Blocks the cycle of lipid metabolic disorder and inflammation	[222]
	Enhances H3K9me3/H3K27me3 modification	Dasatinib + Quercetin	Reduces inflammation and improves cognitive function	[223]
	Enhances H4R3me2as modification	Metformin, Rapamycin, Resveratrol	Regulates SASP and anti - apoptotic genes	[225]
Acetylation	Inhibits HDAC1/2 and enhances H3K9 and H4 acetylation	Trichostatin A (TSA), Suberoylanilide hydroxamic acid (SAHA)	Restores EC - SOD expression and inhibits ROS	[226]
	Inhibits H3K9/14 acetylation	TSA	Anti - fibrotic and inhibits SASP	[227]
	Regulates H3 acetylation	Honokiol (HNK)	Inhibits NF - κB and promotes IL - 10	[228]
	Inhibits HDAC3	Chitinase 1 (CHIT1)	Inhibits neuroinflammation and improves cognition	[229]
	Inhibits HDAC	Valproic acid (VPA)	Alleviates diabetes - related aging and SASP phenotypes	[230]
	Activates SIRT1/SIRT6	SIRT activators	Inhibits COPD immune aging and provides vascular protection	[189, 231]
Ubiquitination	Upregulates CHIP and promotes MYH9 ubiquitination	4,5 - Dicaffeoylquinic acid	Inhibits mitochondria and SASP	[13]
	Upregulates MARCHF3 and promotes NLRP3 ubiquitination	Bushen Huoxue acupuncture	Inhibits pyroptosis and inflammatory factors and improves AD	[234]
	Promotes K48 ubiquitination of p65	Friedelin(FR)	Inhibits NF - κB and alleviates tendon degeneration	[235]
O-GlcNAcylation	Inhibits OGA and enhances O - GlcNAcylation	Thiamet-G	Reduces tau pathology and slows down neuroinflammation	[17]
	Enhances O - GlcNAcylation	Thiamet-G	Enhances the utilization of UDP - GlcNAc	[216]

## Data Availability

Not applicable.
